# Supplementary material for: Global View of Domain-Specific O-Linked Mannose Glycosylation in Glycoengineered Cells
Source: Mol Cell Proteomics. 2024 Jun 6;23(7):100796. doi: 10.1016/j.mcpro.2024.100796 (PMC11292533; doi:10.1016/j.mcpro.2024.100796)
Supplement: supplemental Fig. S1 [file mmc1.pdf]

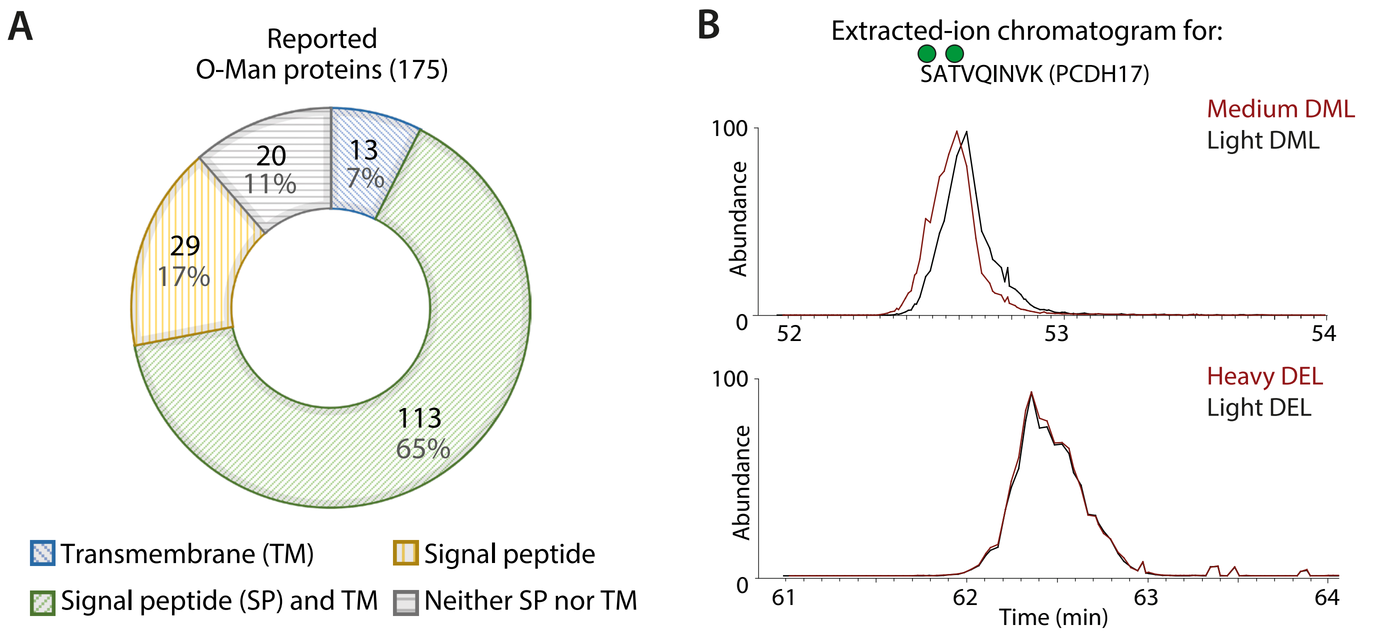
A
Reported
O-Man proteins (175)
13
7%
113
65%
29
17%
20
11%
Transmembrane (TM)
Signal peptide
Signal peptide (SP) and TM
Neither SP nor TM
B
Extracted-ion chromatogram for:
SATVQINVK (PCDH17)
Medium DML
Light DML
100
0
Abundance
52
53
54
Heavy DEL
Light DEL
100
0
Abundance
61
62
63
64
Time (min)
