# Supplementary figures and images for: Global View of Domain-Specific O-Linked Mannose Glycosylation in Glycoengineered Cells
Source: Mol Cell Proteomics. 2024 Jun 6;23(7):100796. doi: 10.1016/j.mcpro.2024.100796 (PMC11292533; doi:10.1016/j.mcpro.2024.100796)

A

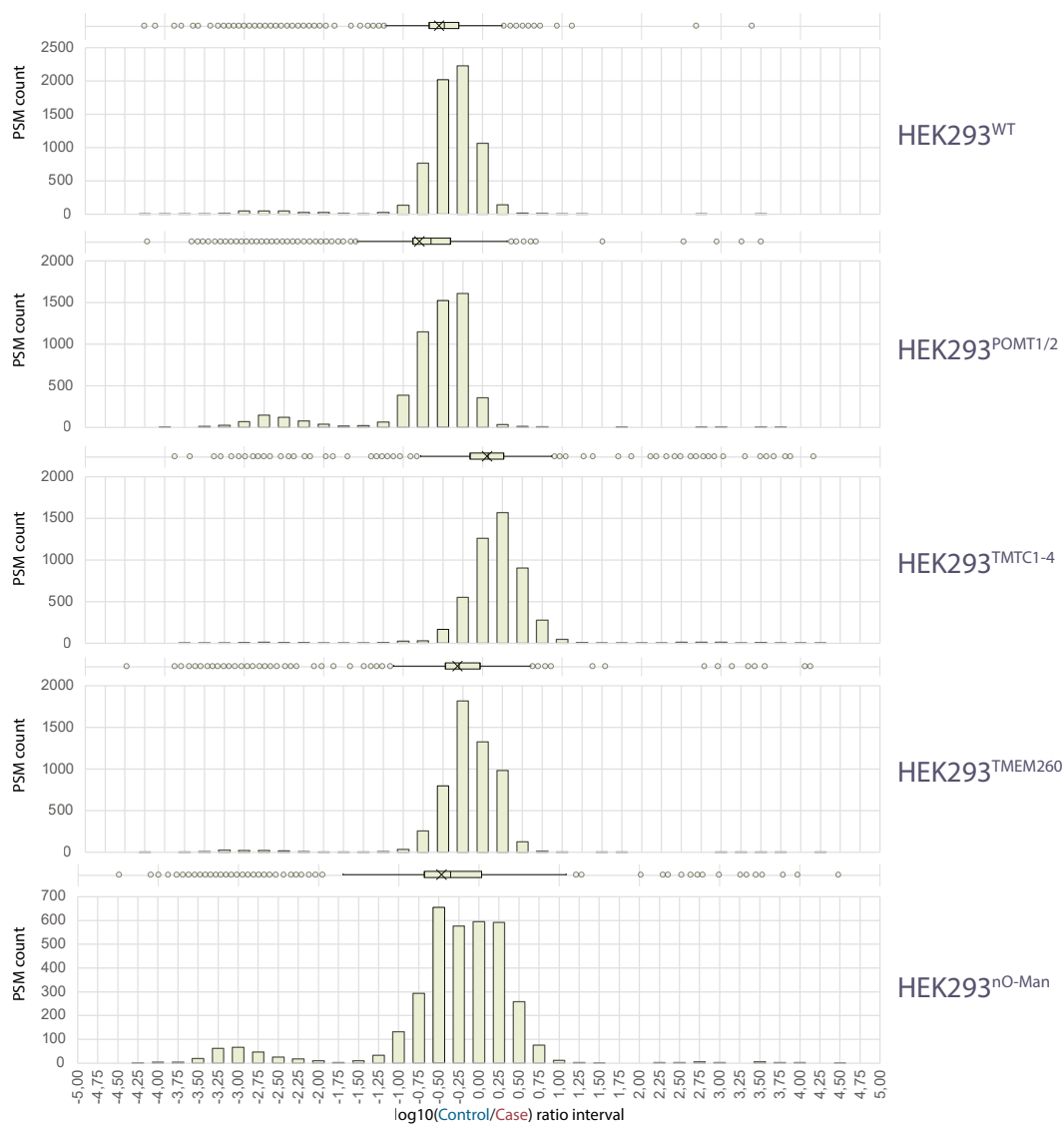

Supplement: supplemental Fig. S2 [file mmc2.pdf]

**A**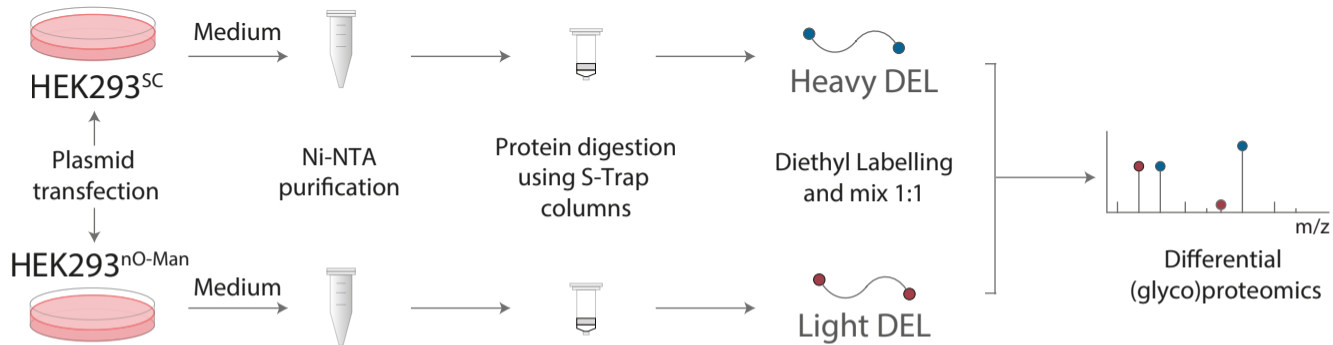**B**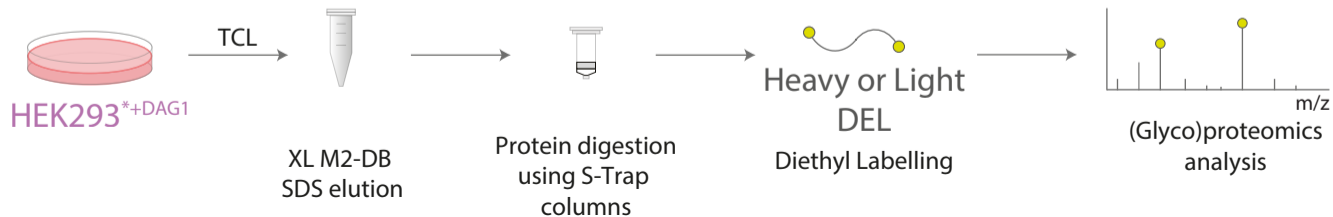

Supplement: supplemental Fig. S4 [file mmc4.pdf]

**A**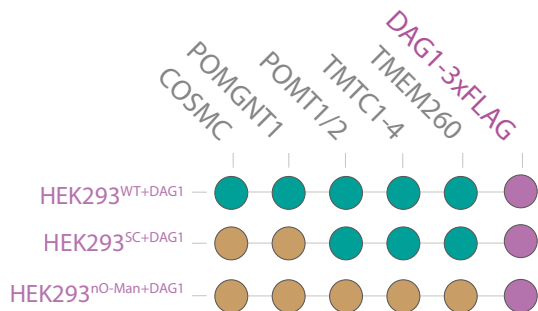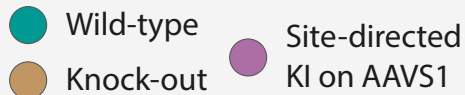

### DAG1-3xFLAG construct

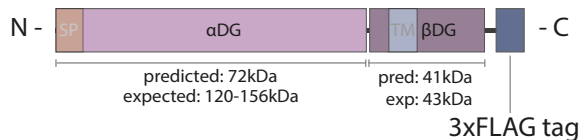**B**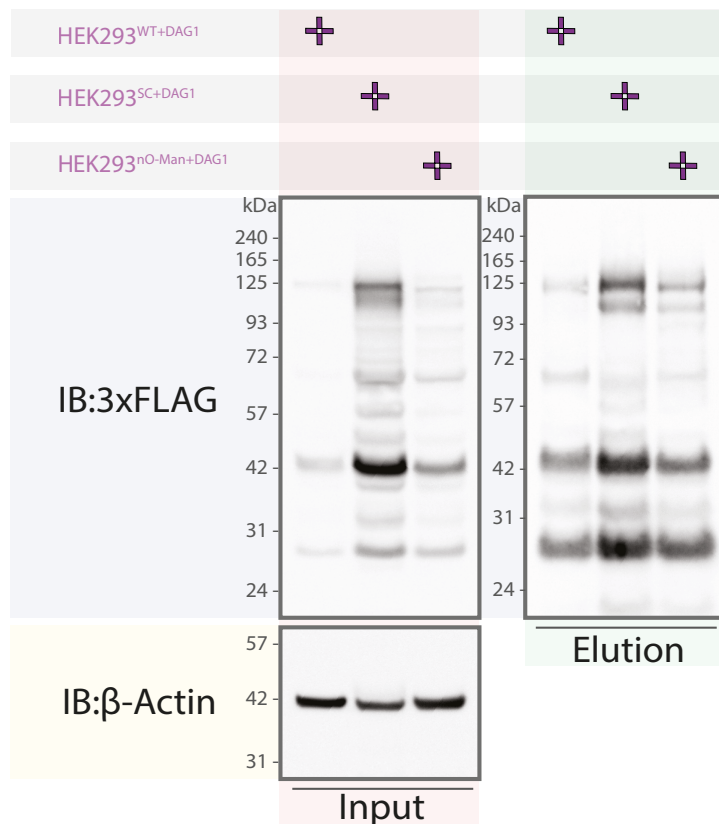

Supplement: supplemental Fig. S5 [file mmc5.pdf]

# A

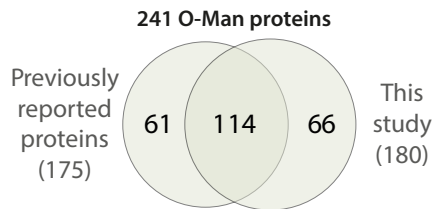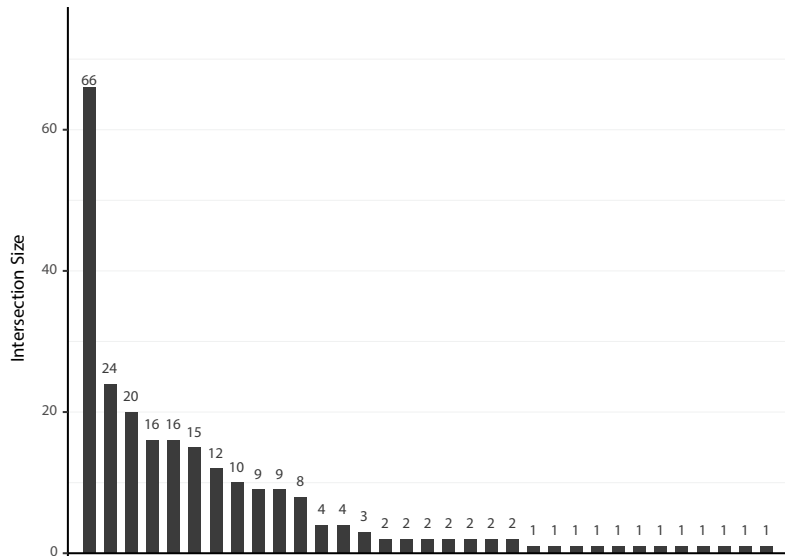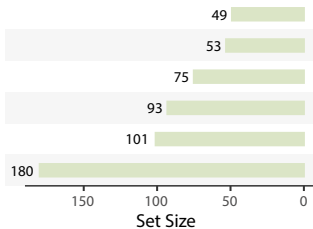

Supplement: supplemental Fig. S6 [file mmc6.pdf]

A

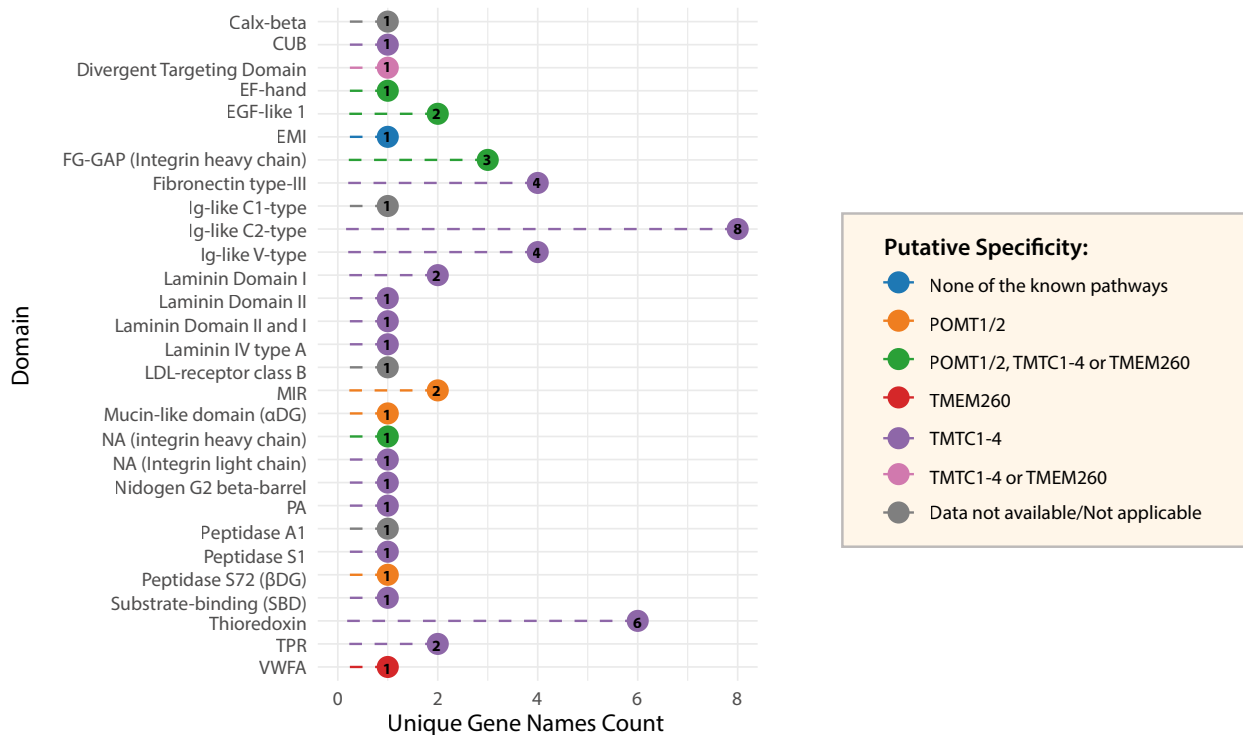

B

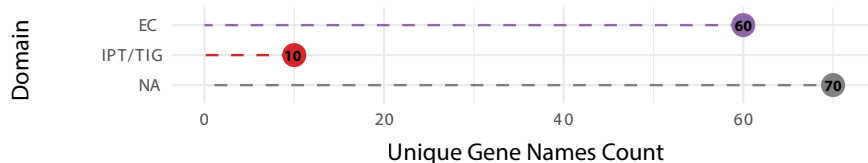

Supplement: supplemental Fig. S7 [file mmc7.pdf]

**A**

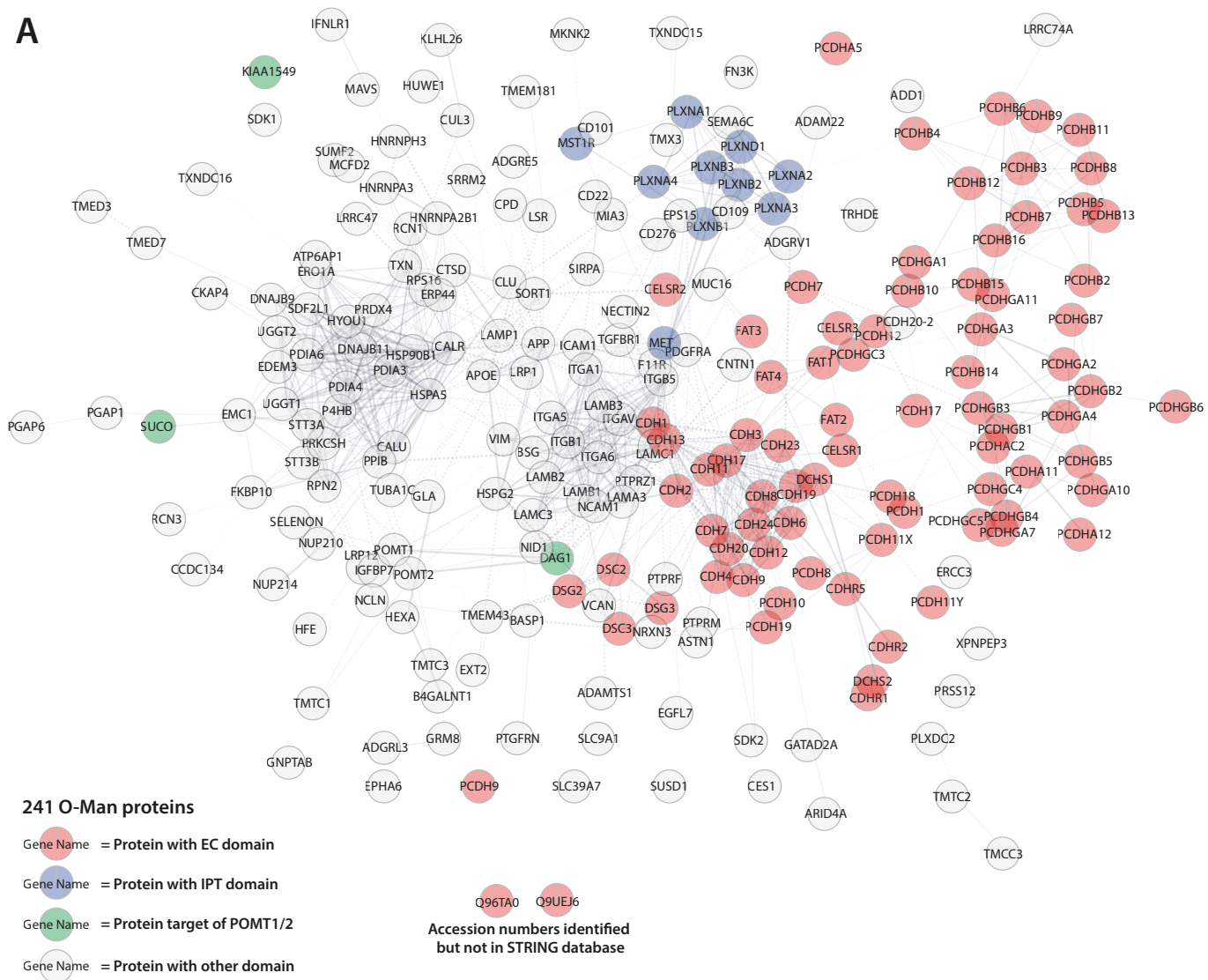

Supplement: supplemental Fig. S8 [file mmc8.pdf]
