# Supplementary material for: Global View of Domain-Specific O-Linked Mannose Glycosylation in Glycoengineered Cells
Source: Mol Cell Proteomics. 2024 Jun 6;23(7):100796. doi: 10.1016/j.mcpro.2024.100796 (PMC11292533; doi:10.1016/j.mcpro.2024.100796)
Supplement: supplemental Fig. S3 [file mmc3.pdf]

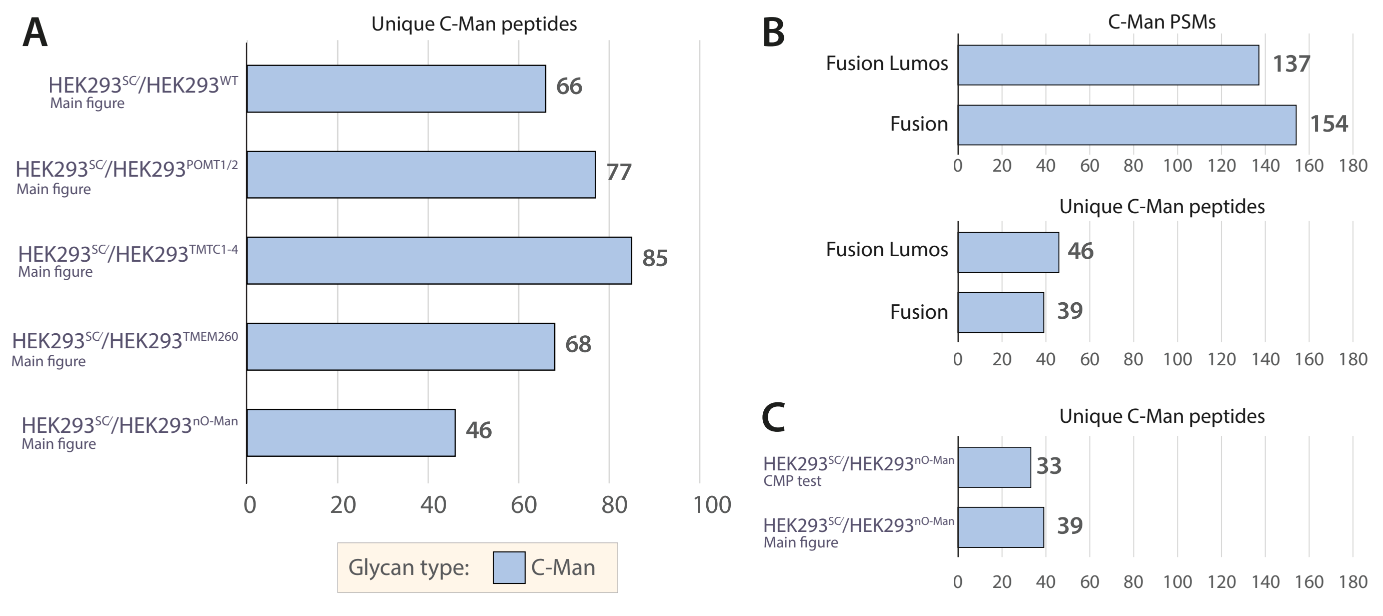
A
Unique C-Man peptides
HEK293SC/HEK293WT
Main figure
66
HEK293SC/HEK293POMT1/2
Main figure
77
HEK293SC/HEK293TMTC1-4
Main figure
85
HEK293SC/HEK293TMEM260
Main figure
68
HEK293SC/HEK293nO-Man
Main figure
46
0
20
40
60
80
100
Glycan type:
C-Man
B
C-Man PSMs
Fusion Lumos
137
Fusion
154
0
20
40
60
80
100
120
140
160
180
Unique C-Man peptides
Fusion Lumos
46
Fusion
39
C
Unique C-Man peptides
HEK293SC/HEK293nO-Man
CMP test
33
HEK293SC/HEK293nO-Man
Main figure
39
