# Supplementary material for: Global View of Domain-Specific O-Linked Mannose Glycosylation in Glycoengineered Cells
Source: Mol Cell Proteomics. 2024 Jun 6;23(7):100796. doi: 10.1016/j.mcpro.2024.100796 (PMC11292533; doi:10.1016/j.mcpro.2024.100796)
Supplement: supplemental Fig. S9 [file mmc9.pdf]

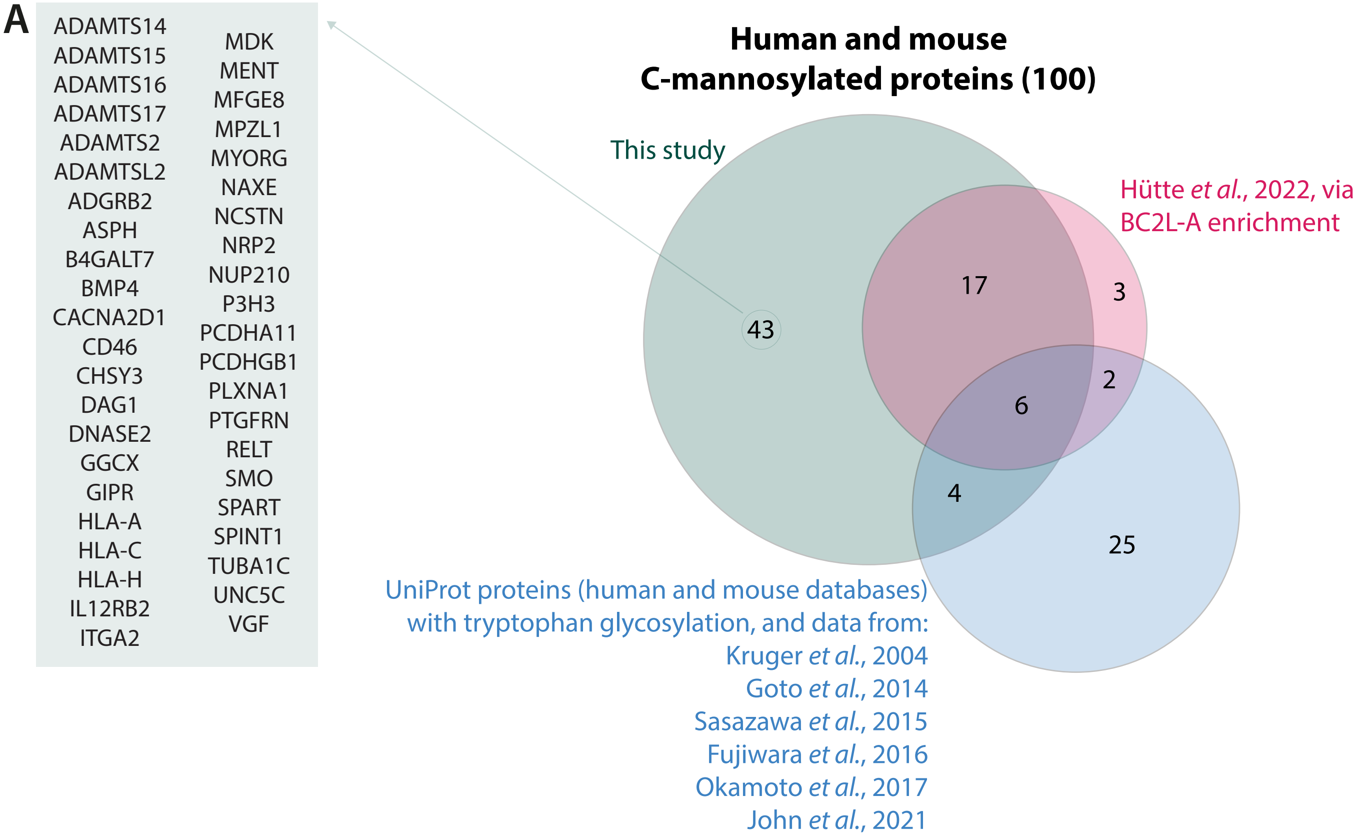
A
ADAMTS14
ADAMTS15
ADAMTS16
ADAMTS17
ADAMTS2
ADAMTSL2
ADGRB2
ASPH
B4GALT7
BMP4
CACNA2D1
CD46
CHSY3
DAG1
DNASE2
GGCX
GIPR
HLA-A
HLA-C
HLA-H
IL12RB2
ITGA2
MDK
MENT
MFGE8
MPZL1
MYORG
NAXE
NCSTN
NRP2
NUP210
P3H3
PCDHA11
PCDHGB1
PLXNA1
PTGFRN
RELT
SMO
SPART
SPINT1
TUBA1C
UNC5C
VGF
Human and mouse C-mannosylated proteins (100)
This study
Hütte et al., 2022, via BC2L-A enrichment
43
17
3
2
6
4
25
UniProt proteins (human and mouse databases) with tryptophan glycosylation, and data from:
Kruger et al., 2004
Goto et al., 2014
Sasazawa et al., 2015
Fujiwara et al., 2016
Okamoto et al., 2017
John et al., 2021
