# Supplementary material for: Global View of Domain-Specific O-Linked Mannose Glycosylation in Glycoengineered Cells
Source: Mol Cell Proteomics. 2024 Jun 6;23(7):100796. doi: 10.1016/j.mcpro.2024.100796 (PMC11292533; doi:10.1016/j.mcpro.2024.100796)
Supplement: supplemental Tables S1–S4 and Figs. S1–S9 [file mmc10.docx]

**Global view of domain-specific O-linked mannose glycosylation in glycoengineered cells**

**Supplementary Data**

Supplementary Data containing individual Datasets 1-36 is uploaded as a separate Excel file.

Identification and quantification of relative abundances of glycopeptides enriched from crude membrane preparations, total cell lysates or from purified proteins. The experimental setup of paired (case and control) analyses of genetically engineered cell lines and their stable isotope coding is described under the "Overview" Sheet. Results from each analysis can be found in individual Sheets (Datasets 1-36).

**Supplementary Tables**

**Supplementary Table 1 – gRNAs**

| Gene name | gRNA Sequence (PAM) | gRNA plasmid ID | Addgene # | Reference |
| --- | --- | --- | --- | --- |
| POMT1 | GAGCTCCAACACTATCTGGT**(AGG)** | Gh23 | 106696 | Described previously in (1, 2) |
| POMT2 | CTTCGAGGCGGTCGGCTGGT**(GGG)** | Gh24 | 106697 | Described previously in (1, 2) |
| TMEM260 | TGGCTATCCTTTGTTCACGC**(TGG)** | Gh231 | N/A | Described previously in (3) |

**Supplementary Table 2 – Primers and oligoes**

| Name | Sequence | Usage | Notes |
| --- | --- | --- | --- |
| DAG1_cDNA_Fwd | 5’ – ATGAGGATGTCTGTGGGCCTCTCGCTGCTG – 3’ | Amplification of DAG1 from HEK293 total cDNA |  |
| DAG1_cDNA_Rev | 5’ - AGGTGGGACATAGGGAGGAGGTGACCGG – 3’ | Amplification of DAG1 from HEK293 total cDNA |  |
| POMT1_IDAA_FwdExt | 5′-AGCTGACCGGCAGCAAAATTGATCAACAGAGCAGCTCCCAT – 3’ | Validation by IDAA of POMT1 KO cells | Described previously in (1) |
| POMT1_IDAA_Rev | 5′-CATGACGGCGCTATGTGAAA – 3’ | Validation by IDAA of POMT1 KO cells | Described previously in (1) |
| POMT2_IDAA_FwdExt | 5′-AGCTGACCGGCAGCAAAATTGCCTGGCAGAGTCCGAGCT -3’ | Validation by IDAA of POMT2 KO cells | Described previously in (1) |
| POMT2_IDAA_Rev | 5′-GACAGCAGCGTCACCAAG – 3’ | Validation by IDAA of POMT2 KO cells | Described previously in (1) |
| TMEM260_IDAA_FwdEext | 5’-AGCTGACCGGCAGCAAAATTGCCATGTGATAGACGCTGCCA-3’ | Validation by IDAA of TMEM260 KO cells | Described previously in (3) |
| TMEM260_IDAA_Rev | 5’-CATGTGTTAGGGAAACCAAGCAA -3’ | Validation by IDAA of TMEM260 KO cells | Described previously in (3) |

**Supplementary Table 3 – Cell lines generated and used**

| Name of the cell line in this study |  | Parental cell line | Reference parental cell line | Modification to parental | INDEL generated and sequence of edited locus (or reference of original study) |
| --- | --- | --- | --- | --- | --- |
| HEK293 or HEK293^WT^ |  | N/A | N/A | N/A | N/A |
| BG1 |  | N/A | N/A | N/A | N/A |
| CaCo-2 |  | N/A | N/A | N/A | N/A |
| HEPG2 |  | N/A | N/A | N/A | N/A |
| SHSY-5Y |  | N/A | N/A | N/A | N/A |
| HEK293^SC^ |  | HEK293^WT^ | N/A | KO of *COSMC* and *POMGNT1* | Described in (1) |
| HEK293^POMT1/2^ |  | HEK293^KO:TMTC1-4^ | (4) | KO of *TMEM260* | TMEM260: +1bp  TGGCTATCCTTTGTT**T**CACGC**TGG** |
| HEK293^TMTC1-4^ |  | HEK293^KO:POMT1, KO:POMT2^ | (1) | KO of *TMEM260* | TMEM260: +1bp  TGGCTATCCTTTGTT**T**CACGC**TGG** |
| HEK293^TMEM260^ |  | HEK293^KO:TMTC1-4^ | (4) | KO of *POMT1* and *POMT2* | POMT1: +2bp GAGCTCCAACACTATC**TG**TGGT**AGG**  POMT2, allele 1: -1bp CTTCGAGGCGGTCGGCT**G**GT**GGG**  POMT2, allele 2: +81bp CTTCGAGGCGGTCGGCT**+81bp**GGT**GGG** |
| HEK293^nO-Man^ |  | HEK293^TMEM260^ | This study | KO of *TMEM260* | TMEM260: +1bp  TGGCTATCCTTTGTT**T**CACGC**TGG** |
| HEK293^WT+DAG1^ |  | HEK293^WT^ | N/A | ZFN KI of DAG1-3xFLAG | N/A |
| HEK293^SC/SC+DAG1^ |  | HEK293^SC/SC^ | (1) | ZFN KI of DAG1-3xFLAG | N/A |
| HEK293^nO-Man+DAG1^ |  | HEK293^nO-Man^ | This study | ZFN KI of DAG1-3xFLAG | N/A |

**Supplementary Table 4 – Proteome Discoverer search parameters**

| Dataset type (Main figure) | Precursor mass tolerance | Static Modifications / +Mass (residue) | Dynamic modifications | Quant |
| --- | --- | --- | --- | --- |
| Standard workflow (Fig. 1) – .raw from (4) | 15 ppm | Carbamidomethyl / +57.021 Da (C)  Dimethyl:2H(4) / +32.056 Da (Any N-Terminus)  Dimethyl:2H(4) / +32.056 Da (K) | Oxidation / +15.995 Da (M)  Hex / +162.053 Da (S, T) | No |
| Improved workflow (Fig. 1) –.raw from Figs. 3, 4, 6 | 15 ppm | Carbamidomethyl / +57.021 Da (C)  Diethyl:[1,2]13C2 / +60.076 Da (Any N-Terminus)  Diethyl:[1,2]13C2 / +60.076 Da (K) | Oxidation / +15.995 Da (M)  Hex / +162.053 Da (S, T) | No |
| WT cells (Fig. 2, Fig. 6) | 10 ppm | Carbamidomethyl / +57.021 Da (C)  Diethyl / +56.063 Da (Any N-Terminus)  Diethyl / +56.063 Da (K) | Oxidation / +15.995 Da (M)  Hex / +162.053 Da (S, T, W) | No |
| Glycoengineered cells (Fig. 1 CMP and TCL, Fig. 3-4, Fig. 6) | 10 ppm | Carbamidomethyl / +57.021 Da (C)  Diethyl:[1,2]13C2 / +60.076 Da (Any N-Terminus)  Diethyl:[1,2]13C2 / +60.076 Da (K)  Diethyl / +56.063 Da (Any N-Terminus)  Diethyl / +56.063 Da (K) | Oxidation / +15.995 Da (M)  Hex / +162.053 Da (S, T, W) | Yes |
| Ratiochecks (Fig. S2) | 15 ppm | Carbamidomethyl / +57.021 Da (C)  Diethyl:[1,2]13C2 / +60.076 Da (Any N-Terminus)  Diethyl:[1,2]13C2 / +60.076 Da (K)  Diethyl / +56.063 Da (Any N-Terminus)  Diethyl / +56.063 Da (K) | Oxidation / +15.995 Da (M) | Yes |
| Soluble model proteins (Fig. 5) | 10 ppm | Carbamidomethyl / +57.021 Da (C)  Diethyl:[1,2]13C2 / +60.076 Da (Any N-Terminus)  Diethyl:[1,2]13C2 / +60.076 Da (K)  Diethyl / +56.063 Da (Any N-Terminus)  Diethyl / +56.063 Da (K) | Oxidation / +15.995 Da (M)  Hex / +162.053 Da (S, T, W)  HexNAc / +203.079 Da (S, T) | Yes |
| Full-length DAG1 (Fig. S4) | 10 ppm | Carbamidomethyl / +57.021 Da (C)  Diethyl:[1,2]13C2 / +60.076 Da (Any N-Terminus)  Diethyl:[1,2]13C2 / +60.076 Da (K)  Diethyl / +56.063 Da (Any N-Terminus)  Diethyl / +56.063 Da (K) | Oxidation / +15.995 Da (M)  Hex / +162.053 Da (S, T, W)  HexNAc / +203.079 Da (S, T) | No |

**Supplementary Figure Legends**

**Figure S1 - Data overview from O-Man glycoproteomic studies and comparison between labelling strategies.** **(A)** 72% of O-Man proteins reported to date are transmembrane proteins and 17% have a signal peptide while only 11% have none of the two, suggesting that most human O-Man proteins are secreted or reside in the secretory pathway. The 175 unique O-Man proteins were extracted from datasets in (1, 3–6), according to Methods section. **(B)** Nano-LC elution profiles of an O-Man glycopeptide labelled with heavy and light DEL (overlapping) and DML (displaced due to deuterium effect).

**Figure S2 – Ratiochecks for differential glycoproteomics samples. (A)** Equal volume of samples labelled with heavy and light DEL were mixed and subjected to proteomics analysis. Calculated ratios (bins) from -5 (minimum value allowed, with log_10_ ratio = 10^-5^) to +5 (maximum value allowed, with log_10_ ratio = 10^5^). "PSM count" represents the frequency of Peptide-Spectrum Matches (PSMs) in each bin. Inter quartile range (IQR) is shown on top of histograms, where “x” depicts the median. The threshold interval for equal abundance of the two channels was set to 10-fold change. Cell line names (right) refer to the nomenclature in *Fig. 3*.

**Figure S3** **– Identification of C-mannosylated peptides in HEK293 cells. (A)** Barchart of unique C-Man glyco-PSMs identified in the five differential glycoproteomics datasets (*Supplementary Data - datasets 15-19*) from O-Man glycoengineered cells. Only data acquired with Fusion Lumos instrument are included in this comparison. **(B)** Barchart of all C-Man PSMs (top) or unique C-Man peptides (bottom) identified from HEK293 SC⁄/HEK293 TMEM260 sample (*Supplementary Data – dataset 19*) analyzed either with Fusion Lumos or Fusion instruments confirms technical replicability of mass spectrometry analysis with different mass spectrometers. **(C)** Barchart of unique C-Man glycopeptides from HEK293^SC^/HEK293^nO-Man^ identified in the TCL/CMP comparison in Figure 1 (*Supplementary Data - dataset 2*) or in Figures 3-4 (*Supplementary Data - dataset 19*), representing biological replicates for the same samples. Only samples analyzed with Fusion mass spectrometer are included in this graph.

**Figure S4 – Schematic representation of workflow for (glyco)proteomics of reporters. (A)** For secreted reporter proteins, glycoengineered cells are transiently transfected with plasmids and media are harvested after 5 days. Ni-NTA purification allows capture of model proteins which are then digested using S-Trap microcolumns and proteases. Peptides are labelled with DEL, mixed 1:1 according to nanodrop measurements (Absorbance = 205 nm) and subjected to MS analysis. **(B)** For full length DAG1, cell pellets from glycoengineered cells stably expressing DAG1-3xFLAG construct are lysed and subjected to capture using M2 antibody conjugated to magnetic beads (XL M2-DB). Proteins are diluted in SDS and digested with trypsin using S-Trap microcolumns. Peptides are labelled with DEL, desalted, and subjected to MS analysis. Genetic backgrounds for the cells used here (in this figure, cumulatively referred to with a star*) are found in Figure S4.

**Figure S5 – Generation and validation of glycoengineered cells expressing full length DAG1. (A)** Overview of DAG1-3xFLAG construct and cell lines generated with KI on AAVS1 locus using zinc finger nucleases. **(B)** Western blot for Input and Elution from anti-FLAG resin confirms expression and enrichment of DAG1 used for proteomics studies as above. Sizes are reported as in (7). The established cell lines show that DAG1-3xFLAG is expressed at different levels.

**Figure S6 – Comparison of O-Man proteins identified in this study and previous glycoproteomics studies. (A)** Of the 180 proteins identified in this study, 66 were not reported in previous glycoproteomics studies (1, 3–6).

**Figure S7 – Protein domains targeted by O-Man glycosylation. (A)** 30 protein domains are identified with O-Man glycosylation. O-Man deconstruction data suggest that TMTC1-4 have capacity to modify several domains and folds, while POMT1/2 and TMEM260 display activity limited to specific protein domains. **(B)** Number of canonical domains identified for TMTC1-4 and TMEM260 pathways. O-Man on unstructured regions on 70 distinct proteins is initiated by either of the three pathways (*cf. Fig. 4A*).

**Figure S8 – Overview of currently known O-Man proteins in humans. (A)** Cumulative visualization of the 241 O-Man proteins identified in this study and in previous glycoproteomics studies (1, 3–6) using STRING according to Methods section. In red are EC domains, in blue IPT domains, in green known POMT1/2 substrates and in gray are proteins with other domains. Few clusters are identified other than these two, and these involve laminins/integrins as well as other ER-resident proteins.

**Figure S9 – Expansion of the C-Man glycoproteome. (A)** This study identified 70 C-Man proteins, 27 of which were previously identified as C-Man proteins in human or mouse cells either with BC2L-A (6) or with other methods (8–13). The Venn diagram depicts number of C-Man proteins identified in this study (green), Hütte et al., 2022 (pink), and entries listed in Uniprot (March 2024) for human (taxonomy_id:9606) or mouse (taxonomy_id:10090) proteins (blue) with carbohydrate modification at Trp residues (ft_carbohyd:tryptophan). The n=43 new C-Man proteins identified in this study are primarily characterized by C-Man on thrombospondin repeats or fibronectin type-III domains (*cf. Fig. 6A*).

**Supplementary References**

(Number of reference according to main text order of appearance is reported after each reference)

1. Larsen, I. S. B., Narimatsu, Y., Joshi, H. J., Yang, Z., Harrison, O. J., Brasch, J., Shapiro, L., Honig, B., Vakhrushev, S. Y., Clausen, H., and Halim, A. (2017) Mammalian O-mannosylation of cadherins and plexins is independent of protein O-mannosyltransferases 1 and 2. *J. Biol. Chem.* **292**, 11586–11598 (Reference number 13)

2. Narimatsu, Y., Joshi, H. J., Yang, Z., Gomes, C., Chen, Y.-H., Lorenzetti, F. C., Furukawa, S., Schjoldager, K. T., Hansen, L., Clausen, H., Bennett, E. P., and Wandall, H. H. (2018) A validated gRNA library for CRISPR/Cas9 targeting of the human glycosyltransferase genome. *Glycobiology*. **28**, 295–305 (Reference number 36)

3. Larsen, I. S. B., Povolo, L., Zhou, L., Tian, W., Mygind, K. J., Hintze, J., Jiang, C., Hartill, V., Prescott, K., Johnson, C. A., Mullegama, S. V, McConkie-Rosell, A., McDonald, M., Hansen, L., Vakhrushev, S. Y., Schjoldager, K. T., Clausen, H., Worzfeld, T., Joshi, H. J., and Halim, A. (2023) The SHDRA syndrome-associated gene TMEM260 encodes a protein-specific O-mannosyltransferase. *Proc. Natl. Acad. Sci. U. S. A.* **120**, e2302584120 (Reference number 18)

4. Larsen, I. S. B., Narimatsu, Y., Joshi, H. J., Siukstaite, L., Harrison, O. J., Brasch, J., Goodman, K. M., Hansen, L., Shapiro, L., Honig, B., Vakhrushev, S. Y., Clausen, H., and Halim, A. (2017) Discovery of an O-mannosylation pathway selectively serving cadherins and protocadherins. *Proc. Natl. Acad. Sci. U. S. A.* **114**, 11163–11168 (Reference number 17)

5. Vester-Christensen, M. B., Halim, A., Joshi, H. J., Steentoft, C., Bennett, E. P., Levery, S. B., Vakhrushev, S. Y., and Clausen, H. (2013) Mining the O-mannose glycoproteome reveals cadherins as major O-mannosylated glycoproteins. *Proc. Natl. Acad. Sci. U. S. A.* **110**, 21018–23 (Reference number 19)

6. Hütte, H. J., Tiemann, B., Shcherbakova, A., Grote, V., Hoffmann, M., Povolo, L., Lommel, M., Strahl, S., Vakhrushev, S. Y., Rapp, E., Buettner, F. F. R., Halim, A., Imberty, A., and Bakker, H. (2022) A Bacterial Mannose Binding Lectin as a Tool for the Enrichment of C- and O-Mannosylated Peptides. *Anal. Chem.* **94**, 7329–7338 (Reference number 40)

7. Barresi, R., and Campbell, K. P. (2006) Dystroglycan: from biosynthesis to pathogenesis of human disease. *J. Cell Sci.* **119**, 199–207 (Reference number 23)

8. Kruger, R. P., Lee, J., Li, W., and Guan, K.-L. (2004) Mapping netrin receptor binding reveals domains of Unc5 regulating its tyrosine phosphorylation. *J. Neurosci.* **24**, 10826–34 (Reference number 54)

9. Goto, Y., Niwa, Y., Suzuki, T., Dohmae, N., Umezawa, K., and Simizu, S. (2014) C-mannosylation of human hyaluronidase 1: possible roles for secretion and enzymatic activity. *Int. J. Oncol.* **45**, 344–50 (Reference number 55)

10. Sasazawa, Y., Sato, N., Suzuki, T., Dohmae, N., and Simizu, S. (2015) C-mannosylation of thrombopoietin receptor (c-Mpl) regulates thrombopoietin-dependent JAK-STAT signaling. *Biochem. Biophys. Res. Commun.* **468**, 262–268 (Reference number 56)

11. Fujiwara, M., Kato, S., Niwa, Y., Suzuki, T., Tsuchiya, M., Sasazawa, Y., Dohmae, N., and Simizu, S. (2016) C-mannosylation of R-spondin3 regulates its secretion and activity of Wnt/β-catenin signaling in cells. *FEBS Lett.* **590**, 2639–49 (Reference number 57)

12. Okamoto, S., Murano, T., Suzuki, T., Uematsu, S., Niwa, Y., Sasazawa, Y., Dohmae, N., Bujo, H., and Simizu, S. (2017) Regulation of secretion and enzymatic activity of lipoprotein lipase by C-mannosylation. *Biochem. Biophys. Res. Commun.* **486**, 558–563 (Reference number 58)

13. John, A., Järvå, M. A., Shah, S., Mao, R., Chappaz, S., Birkinshaw, R. W., Czabotar, P. E., Lo, A. W., Scott, N. E., and Goddard-Borger, E. D. (2021) Yeast- and antibody-based tools for studying tryptophan C-mannosylation. *Nat. Chem. Biol.* **17**, 428–437 (Reference number 59)
